# Preparation and characterization of gelatin/chitosan nanocomposite reinforced by NiO nanoparticles as an active food packaging

**DOI:** 10.1038/s41598-023-50260-8

**Published:** 2024-01-04

**Authors:** Mahdieh Momtaz, Elham Momtaz, Masoud A. Mehrgardi, Fatemeh Momtaz, Tahmineh Narimani, Farkhondeh Poursina

**Affiliations:** 1 Department of Microbiology, School of Medicine, Isfahan University of Medical Sciences, 81746-73461, Isfahan, Iran; 2 Department of Chemistry, University of Isfahan, Isfahan, 8174673441 Iran

**Keywords:** Antimicrobials, Polymer chemistry

## Abstract

Food packaging with antibacterial properties has attracted much attention recently. In this study, nickel oxide nanoparticles (NiONPs) were synthesized by co-precipitation and then gelatin/chitosan polymer films (GEL/CS) with different percentages of NiONPs, bio-nanocomposites, were prepared by casting. Morphology, crystal microstructure, molecular interactions and thermal stabilities of the NPs and the composite films were characterized by FESEM, XRD, FTIR and TGA, respectively. The bio-nanocomposite films exhibited excellent barrier, thermal and mechanical properties by addition of an optimized content of NPs. For example, the tensile strength (TS) of the GEL/CS film without NPs was 23.83 MPa and increased to 30.13 MPa by incorporation of 1% NPs. The antibacterial properties and toxicity of the films were investigated. These films show good antibacterial behavior against Gram-positive (*Staphylococcus aureus*) bacteria compared to Gram-negative (*Escherichia coli*) bacteria. Furthermore, the films were found to be non-toxic to fibroblast cells that came into contact with the films, with a survival rate of more than 88%. Therefore, these films can be applied for food packaging due to their excellent mechanical, barrier, and antibacterial properties.

## Introduction

Food packaging plays an important role in protecting food from chemical, biological and physical hazards^1^. Petroleum-based plastic polymers are widely used due to their desirable properties such as good barrier effect, high tensile strength, light weight and relatively low price^2^, but they generate a lot of waste in the environment due to their indestructibility and very long shelf life^3^. Therefore, the food industry is looking for antibacterial and biocompatible packaging materials that extend the shelf life of food^4^ and provide a suitable alternative to non-degradable petroleum-based plastics. For this reason, natural polymers such as proteins (gelatin) and polysaccharides (chitosan) are used nowadays^5^. Chitosan is a linear polysaccharide with D-glucosamine and N-acetyl-D-glucosamine units linked by β-(1-4) glycosidic bonds. Chitosan is obtained from the deacetylation of chitin, which is extracted from the skin of crustaceans such as crabs and shrimp in an alkaline solution. During the deacetylation process, the acetyl group in the chitin molecule is replaced by the amino group. Chitosan, with its biodegradability, biocompatibility, low toxicity, excellent film formation, availability, cationic nature and inherent antimicrobial properties, has attracted great interest in the food packaging industry^6^. Chitosan films are limited due to their weak mechanical and barrier properties, which can be improved by creating hydrogen bonds between the hydroxyl groups of chitosan and natural polymers, including gelatin, via the amine groups^7^. Gelatin is a water-soluble animal protein derived from the partial hydrolysis of collagen in the fibrous tissues of animals (e.g., cattle, pigs, and fish). As a biocompatible polymer with excellent film-forming properties that is ubiquitous and cheap, gelatin is widely used in the food packaging industry^8,9^. This natural polymer with amphoteric properties is composed of various amino acids. These negatively charged amino acids of gelatin can form strong electrostatic interactions with the positively charged amino acids of chitosan to form films^10^. However, gelatin/chitosan composite films have poor mechanical, thermal, barrier, and antibacterial properties, and the addition of metal oxide NiONPs as fillers to this bio-composite improves the film properties^11–13^. NiONPs remain one of the most important metal oxides, which have received much attention due to their high thermal and chemical stability, large surface area, low price and ubiquity^14^. Moreover, researchers have used NiONPs as one of the fillers and reinforcing agents in biopolymers due to their antibacterial properties, non-toxicity and environmental friendliness^15^. Therefore, many researches have been conducted in the field of studying the antibacterial properties of nickel oxide in combination with polymers^16–19^. However, to our knowledge, no studies have been conducted on GEL/CS biocomposite films containing NiONPs; this objective was achieved for the first time in the present study. Accordingly, GEL/CS polymer films were prepared with different amounts of NiONPs (0.5%, 1%, and 2%) by casting method. Then, their morphological, antibacterial, mechanical, thermal and toxic properties were analyzed. This analysis aims to show how the addition of NPs can improve the antibacterial defense, mechanical strength and barrier properties of biocomposite films, making them suitable for food packaging.

## Results and discussion

### Nanoparticles characteristics

**Figure 1:**
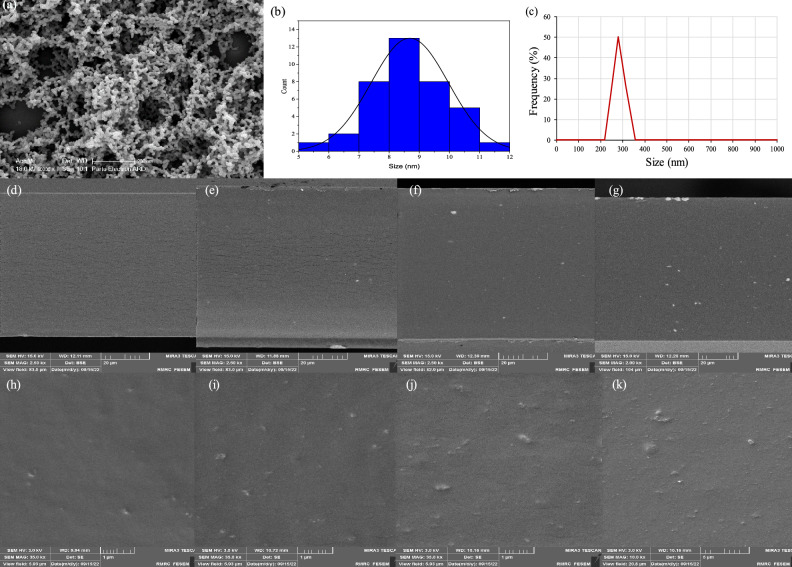
SEM image of synthesized NiONPs (**a**), size distribution histogram (**b**), the average size of NiONPs with the polydispersity index (PDI) (**c**) and images of the cross-section surface of the bio-nanocomposite films involving 0% (**d**), 0.5% (**e**), 1% (**f**), 2% (**g**), 0.5% (**h**), 1% (**i**), 2% (**j**) and 2% of the NiONPs using FESEM technique (**k**).

The SEM image and size distribution histogram of the synthesized material are shown in Fig. 1(a and b). As can be clearly seen in Fig. 1a, the synthesized NiONPs have a uniform and dense spherical shape. According to the histogram (Fig. 1b), the average size of the particles is 8.6 ± 1.2 nm. These results are comparable to those reported by *Deshpande et al.*^20^ confirming a true synthesis of the NPs. Dynamic light scattering (DLS) method is usually used to monitor poly dispersity index (PDI) of nanoscale particles. The data showed a mean hydrodynamic particle diameter of 264±22 nm and a PDI of 0.0074 (Fig. 1c). The DLS results indicate that the nanoparticles are monodisperse in the aqueous medium.

### Film properties

The properties of the bio-nanocomposite films were investigated in terms of particle size distribution, particle dispersion and cross-sectional morphology of the film using a FESEM technique. Figure 1(d–g) shows images of the cross sections of GEL/CS and GEL/CS films containing different percentages of NiONPs, e.g., 0.5%, 1.0%, and 2.0%, respectively. As can be seen, the cross-sectional morphology of the GEL/CS film is smooth and homogeneous with small cracks, indicating very good mixing and chemical bonding between GEL and CS macromolecules (Fig. 1d). The particle size distribution in the films GEL/CS/NPs 0.5% and GEL/CS/NPs 1% is 40 ± 5 and 42 ± 5 nm, respectively, indicating monodispersion of NPs in the synthesized bio-nanocomposites. These NPs are individually and uniformly distributed in the film without aggregating (Fig. 1h and i), which hinders the permeability of water molecules. The addition of NiONPs (up to 1%) increased the contraction and density of the films by reducing the voids, as NiONPs can balance the intermolecular gap between the components of the GEL/CS mixture, indicating an attractive interaction between the NPs and GEL/CS, which makes the composite more desirable in terms of mechanical properties, WVP, MA and antibacterial properties. Increasing the percentage of NPs (e.g., 2%) increased the average size of NPs. Thus, on the image of the cross-sectional area of the film (Fig. 1j), a small accumulation of the NPs with an average size distribution of 52 ± 4 nm can be seen. These nanoparticles are highly accumulated and deteriorate the mechanical properties of the bio-nanocomposite. In Fig. 1k, larger agglomerations of NPs can also be seen on the FESEM image of the film. The aggregation of NPs according to their percentage within the polymers was also described by *D. Lin* and *S. Marand*^19,21^. The PDI of NiO nanoparticles in GEL/CS mixtures was determined by DLS. The PDI of GEL/CS/NPs 0.5%, GEL/CS/NPs 1% and GEL/CS/NPs 2% combinations was 0.0094, 0.0098 and 0.0153, respectively. In all cases, the measured PDI was low (PDI < 0.1), indicating monodispersion of the nanoparticles in the mixture.

### FTIR spectra

**Figure 2:**
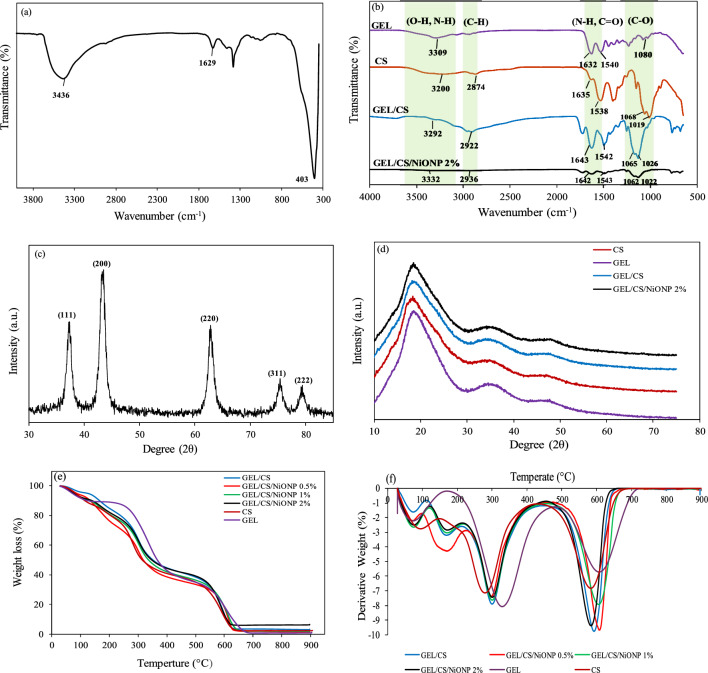
The FTIR spectrum of NiONPs (**a**); different compositions of films (**b**); XRD patterns of NiONPs (**c**); different types of the prepared films (**d**); TGA thermograms and DTG curves of films (**e**, **f**).

FTIR spectra were recorded to find out and analyze the molecular interactions within chitosan, gelatin, GEL/CS, GEL/CS/NPs 2% films, and NiONPs. These spectra are shown in Fig. 2(a and b). In the FTIR of the NPs, the broad absorption peak of 3436 cm^−1^ is associated with the O–H stretching vibration of interlayer water molecules, and the weak peak of 1629 cm^−1^ is attributed to the H–O–H bending vibration of the water molecules absorbed at the surface of the synthesized nickel oxide structure^22,23^. The most intense absorption peak around 403 cm^−1^ is the fingerprint of the metal oxide structures that are NiONPs in this study^24^. According to the structural features of the chitosan polymer, the broad weak peak is at 3200 cm^−1^, the narrow weak peak is at 2874 cm^−1^, the peak is at 1635 cm^−1^, the peak is at 1538 cm^−1^, and the peaks at 1019 and 1068 cm^−1^ belong to the stretching vibrations of the O–H and N–H groups, the C–H bond, the amide I group, the amino N–H group (amide II), and the C–O bond, respectively. The peak of 1410 cm^−1^ can also be assigned to the bending vibration mode of the group CH^25–28^. FTIR characterization of the gelatin film shows that the broad weak peak around 3309 cm^−1^ belongs to the N–H stretching vibration of amide A, the peak around 1632 cm^−1^ belongs to the stretching C=O group of amide I, the peak at 1540 cm^−1^ belongs to the bending N–H group of amide II and stretching C–N bond, the peak at 1235 cm^−1^ belongs to the stretching vibration mode of N–H and C–N groups (amide III) and the fine peak at 1080 cm^−1^ belongs to the stretching C–O bond^25,26,29^. The spectrum of the GEL/CS composite shows shifts in some specific absorbances due to the interactions between the amine and carboxylic acid groups of chitosan and gelatin, respectively. For example, in the FTIR spectrum of the pure gelatin film, the peak at 1540 cm^−1^ (N–H amide II) and the peak at 1632 cm^−1^ (C=O carbonyl and amide I) have shifted to the peaks at 1542 cm^−1^ and 1643 cm^−1^, respectively, in the FTIR spectrum of GEL/CS. Therefore, the changes in the carbonyl (amide I) and amine (amide II) peaks of chitosan and gelatin indicate the formation of new hydrogen bonds between these functional groups of gelatin and chitosan in the GEL/CS mixture. Moreover, the peak around 1538cm^−1^ of the amide group II in chitosan shifted to 1542 cm^−1^ in the GEL/CS mixture, confirming the presence of an electrostatic interaction between the carboxyl group of gelatin and the amino group of chitosan. As shown by the FTIR analysis of the bio-nanocomposites (Fig. 2b), the characteristic peaks of the chitosan and gelatin polymers in the GEL/CS/NiONPs 2% bio-nanocomposite were attenuated by the presence of NiONPs. The absorption peak related to the stretching of the O–H group shifted slightly from 3292 cm^−1^ in GEL/CS to 3332 cm^−1^ in GEL/CS/NiONPs 2%, indicating the addition of NiONPs. Since the NiONPs are rich in OH groups, the NPs can interact with GEL/CS by forming hydrogen bonds, resulting in a shift of the characteristic peak of the OH group to larger wavenumbers. As a result, changes in the spectra belonging to hydroxyl, amine and amide groups attached to GEL/CS/NiONPs 2% bio-nanocomposites reveal the chemical interactions between NiONPs and GEL/CS polymers.

### XRD patterns

To unravel the microstructural features of the synthesized structures, the X-ray diffraction patterns of NiONPs, pure gelatin, pure chitosan, GEL/CS mixture and GEL/CS/NiONPs 2% were recorded and shown in Fig. 2(c and d). In the XRD pattern of the pure gelatin and chitosan films, a sharp peak can be seen at 2θ = 19.1° and 2θ = 19.0°, respectively, confirming the amorphous structure of the polymers. This phenomenon can be attributed to a large number of hydroxyl (–OH) and amine (–NH_2_) groups attached to chitosan molecules and forming hydrogen bonds^30–32^. Addition of gelatin to chitosan shifts this amorphous peak to 2θ = 19.1° and it appears with lower intensity, indicating a strong interaction between chitosan and gelatin. In the X-ray diffraction pattern of NiONPs, a series of peaks occurred at 37.2°, 43.27°, 62.86°, 75.39° and 79.38°, which are related to the planes of (111), (200), (220), (311) and (222), respectively, according to the Joint Committee on Powder Diffraction Standards (ICDD). This diffraction pattern is assigned to the cubic structure of bunsenite (ICDD PDF No.01-071-1179). Based on the XRD pattern of NiONPs, the purity of the synthesized nickel oxide crystals can be demonstrated^20,22^. The addition of 2% NPs to the GEL/CS mixture does not show a specific peak of NiONPs in the XRD pattern of GEL/CS/NiONPs 2%, but the intensity of the peak became slightly sharper at 2θ = 19.2°, which can be attributed to the presence of NiONPs and their effects on the microstructure of the composite. The extremely low percentage of NPs, less than 5%, has resulted in the absence of the characteristic peaks of NiONPs.

### Thermal analysis

The thermal stability of nanocomposite films used for food packaging is one of the most important properties to be investigated and reported accordingly. The TGA/DTG of gelatin, chitosan, GEL/CS and GEL/CS/NiONPs (0.5%, 1.0% and 2.0%) is shown in Fig. 2(e,f). As this figure illustrates, chitosan is decomposed in three stages, the first stage being weight loss at 80–115 °C due to the removal of moisture. The second stage of weight loss at a temperature of 260–300 °C is due to the decomposition of polysaccharides, and the final stage of weight loss in the range of 560–600˚C is due to the destruction of carbons. Similarly, gelatin was decomposed in three stages as well. The first stage of weight loss takes place at a temperature of 50–100 °C and is due to the removal of moisture. The second stage of weight loss is at a temperature of 300–350 °C, which is due to the decomposition of proteins, and finally, at a temperature in the range of 580–630 °C, the remaining materials of the composition were destroyed. DTG curves and TGA thermogram show that the thermal stability of gelatin is higher than that of chitosan, and by adding gelatin to chitosan, the thermal stability of GEL/CS is higher than that of chitosan itself. In addition, the thermal stabilities of bionanocomposite films were investigated. The films were decomposed in four main stages, which are mainly described as evaporation of moisture and decomposition of the different components of the film. The first stage is related to water loss at 60–120 °C. The second phase of weight loss occurs at 170–215 °C and is related to the evaporation of water molecules absorbed in the small pores and to the decomposition of glycerol. In the third stage, at 250–400 °C, there is a significant reduction in weight, which is an expression of the denaturation and depolymerization of the macromolecules in the composite. The final stage occurs at temperatures above 500 ˚C, where the organic components (e.g., chitosan and gelatin) of all samples of the bio-nanocomposite decompose rapidly and exhibit a similar rate of decomposition^24,33^. It was found that the different percentages of NiONPs in the GEL/CS mixture slightly increase the thermal stability and degradation temperature of the GEL/CS nanocomposites, which is mainly due to the chemical interactions between the polymer strands that prevent the degradation of the polymer mixture. Therefore, these films are thermally stable and suitable for food packaging.

### Mechanical properties

The mechanical strength of composite films is a critical criterion for determining integrity resistance under harsh environmental conditions, such as those that may be encountered in packaging. Two valuable parameters for evaluating the mechanical strength of composite films are tensile strength (TS) and elongation at break (EAB), which are listed in Table 1. In this table, the values of TS and EAB for the films GEL/CS (1.0% and 2.0%) and GEL/CS/NiONPs (0.5%, 1.0% and 2.0%) are compiled for comparison. The change in mechanical properties of films is generally due to the interaction between their components. As shown in this table, an increase in the chitosan content in the film GEL /CS significantly (*P* ˂ 0.05) affects the parameters TS and EAB of the film. The parameters TS and EAB of the films increased with the increase of the chitosan content, which can be attributed to the increase of the binding interactions between the ingredient molecules of the film mixture^34^. Due to the better TS and EAB values for GEL/CS 2% compared to GEL/CS 1% film from now on other tests including antibacterial and all food packaging tests are reported for GEL/CS 2% film with different percentages of NiONPs. The TS and EAB in Table 1 show that the addition of NPs up to 1% significantly (P ˂ 0.05) increases the mechanical strength of the films compared with the control film, which may be due to the new intermolecular binding interactions between GEL/CS and NiONPs in the bio-nanocomposite polymer^35^. The significant reduction (P ˂ 0.05) of TS and EAB in GEL/CS/NiONPs 2% film compared with the control film can be explained by the accumulation of NPs in the polymer mixture, which could be caused by a reduction in the binding interactions between GEL/CS chains and NPs. In general, the addition of NPs in low percentages can effectively improve the mechanical properties of films, while too many NPs within the film texture make the films more brittle and fragile, which is consistent with the literature^36,37^.

**Table 1:** The WVP, MA, WS and mechanical properties of the bio-nanocomposite films.

Sample	WVP (g mm/m^2^ day kPa)	MA (%)	WS (%)	EAB (%)	TS (MPa)	Thickness (mm)
GEL/CS 1%	–	–	–	16.84 ± 1.04^a^	12.32 ± 0.37^a^	0.04
GEL/CS 2%	0.62 ± 0.04^a^	7.25 ± 0.92^a^	23.68 ± 0.34^a^	22.53 ± 0.72^b^	23.83 ± 1.09^b^	0.04
GEL/CS/NiONPs 0.5%	0.55 ± 0.03^b^	6.07 ± 0.88^a^	22.46 ± 0.94^b^	26.43 ± 1.03^c^	26.45 ± 1.43^c^	0.04
GEL/CS/NiONPs 1%	0.47 ± 0.02^c^	3.68 ± 0.38^b^	19.11 ± 0.17^c^	26.99 ± 1.24^c^	30.13 ± 0.70^d^	0.04
GEL/CS/NiONPs 2%	0.49 ± 0.03^b,c^	3.90 ± 0.99^b^	21.02 ± 0.67^d^	20.00 ± 0.50^d^	26.61 ± 2.00^c^	0.04

Values are presented as mean ± standard deviation (n = 3). Different letters in the same column indication significant differences (*P* < 0.05).

### Water resistance properties

#### Water vapor permeability

Water vapor permeability (WVP) testing is an essential test to ensure the resistance of packaging films to the diffusion of water molecules. Moisture exchange between the inside (e.g., all materials inside the package) and the outside (e.g., the environment outside the package) of a given package is undesirable in food packaging. This moisture-insulating property of the packaging films helps to maintain the quality of the food over a longer period of time. Table 1 shows the WVP results of the film samples, including GEL/CS 2.0% and GEL/CS/NiONPs (0.5%, 1.0% and 2.0%). As can be seen from this table, the control film GEL/CS 2.0% is more permeable to moisture than the other films with NiONPs. Furthermore, for the films with NPs, increasing the NP content to 1.0% decreases the WVP, while for the GEL/CS/NiONPs film with 2.0%, the WVP increases. This observation can be illustrated by the fact that the addition of NPs up to 1% makes the composite more compact by strengthening the hydrogen bonds between the polymer components; however, more NPs lead to agglomeration and non-uniform distribution of NPs, which increases the voids in the polymer and leads to more WVP of the film^12,21^.

#### Moisture absorption

Moisture absorption (MA) is one of the fundamental properties of biocompatible nanocomposite films for food packaging. This is because films that absorb less moisture are more reliable for food preservation. The results of MA tests of GEL/CS film (control film) and nanocomposite films with different percentages of NPs are shown in Table 1. From this table, it can be seen that the control film has the highest MA of 7.25% after 5 days, while the other samples containing NPs have significantly less (*P* ˂ 0.05) MA compared to the control film. This behavior can be attributed to two facts: first, the filling of the voids of the polymer with NPs and, second, the formation of strong bonds between the components of the composite in the presence of the NPs due to the formation of cross-links and new hydrogen bonds, which reduce the mobility of the chains accordingly. These observations and explanations have been repeated in the literature for the mixtures of zinc oxide NPs with cellulose fibers^38^ and NiONPs with chitosan^19^.

#### Water solubility

Biocompatible polymers used for food packaging must be as insensitive to moisture as possible, since their high water solubility is not desirable in practice. For this reason, the WS tests were conducted in this study. A major drawback of biopolymers is their sensitivity to moisture compared to synthetic polymers; for this reason, recent studies have considered increasing the water resistance of biopolymers. The WS results of the control film and nanocomposite film samples containing different percentages of NiONPs are shown in Table 1. The addition of NPs with percentages of 0.5 and 1% decreases the WS compared to the control film, while in the nanocomposite film with 2% NPs, the WS remains higher than that of the 1% NPs. It appears that the addition of NiONPs to the polymer mixture initially decreases WS due to strong crosslinking between the nanocomposite constituents and consequently imposing some limitations on the chain movements^39^. However, this scenario is reversed by the addition of 2.0% NPs, which can be explained by two reasons. First, NiONPs accumulated and their dissolution was facilitated. Second, these accumulated NPs cause less hydrogen bonding between GEL/CS and NiONPs^12^.

### Antimicrobial properties

Food spoilage is the direct result of the proliferation and growth of microorganisms inside the packaging, and this problem could be regulated by the use of antibacterial packaging films. Therefore, it seemed necessary to investigate the antibacterial activity of the packaging films produced in this study. The disk diffusion method was used to evaluate the antibacterial activity of the films prepared in this study. In this assay, food contaminating indicator strains of Gram-positive (*Staphylococcus aureus*) and Gram-negative (*Escherichia coli*) bacteria were analyzed against GEL/CS (control) and GEL/CS/NiONPs (0.5%, 1.0%, and 2.0%) bio-nanocomposite films. These strains usually attack vegetables and cause them to decompose and rot. The zone of inhibition around the GEL/CS film against *Staphylococcus aureus* and *Escherichia coli* was observed due to the inherent antimicrobial activity of chitosan. The antimicrobial property of chitosan is due to the electrostatic interaction between the positively charged amino groups on chitosan and the negatively charged cell membrane, which leads to the leakage of proteins and other components of microorganisms such as bacteria and ultimately prevents their growth^40,41^. In addition to the inherent antibacterial activity of chitosan, its synergistic effect with NiONPs causes a significant increase (P ˂ 0.05) in the antibacterial activity of the films with NPs compared to the control film, as can be inferred from the results listed in Table 2 and also reported in the literature^42^. The antibacterial activity of NiONPs is explained by three different mechanisms, including the activation of the apoptosis process due to the interaction between the NPs and the bacterial surface, the damage to the cell membrane caused by the released Ni^+2^ from the NiONPs and the interaction with the negatively charged membrane of the bacteria, and the oxidative stress of the cell due to the production of reactive oxygen species (ROS), which causes DNA damage, protein denaturation, and mitochondria damage, ultimately leading to cell death^15,43^. In the present study, NiONPs in a biocompatible composite were shown to have better antibacterial activity against Gram-positive bacteria than against Gram-negative bacteria. Gram-negative bacteria possessing a negatively charged double layer, single-layer membrane with thin peptidoglycan and lipopolysaccharide are less permeable to ROS and Ni^+2^ ions, while Gram-positive bacteria with a less negatively charged thick multilayered membrane are more sensitive to the antibacterial effect of NiONPs. It is worth noting that the actual mechanism of this behavior is unclear and still under investigation^19^. Due to the excellent antibacterial properties of the prepared bio-nanocomposite GEL/CS/NiONPs, it can be introduced as a good candidate for food packaging.

**Table 2:** Antimicrobial activity of bio-nanocomposite films.

Samples	Inhibitory zone (mm)	
	*S. aureus*	*E. coli*
GEL/CS (Control)	9.00 ± 0.20^a^	9.00 ± 0.35^a^
GEL/CS/NiONPs 0.5%	10.50 ± 0.06^b^	10.00 ± 0.15^b^
GEL/CS/NiONPs 1%	13.00 ± 0.30^c^	10.00 ± 0.06^b^
GEL/CS/NiONPs 2%	14.00 ± 0.15^d^	11.00 ± 0.31^c^

Values are presented as mean ± standard deviation (n = 3). Different letters in the same column indication significant differences (*P* < 0.05).

### Cytotoxicity evaluation

**Figure 3:**
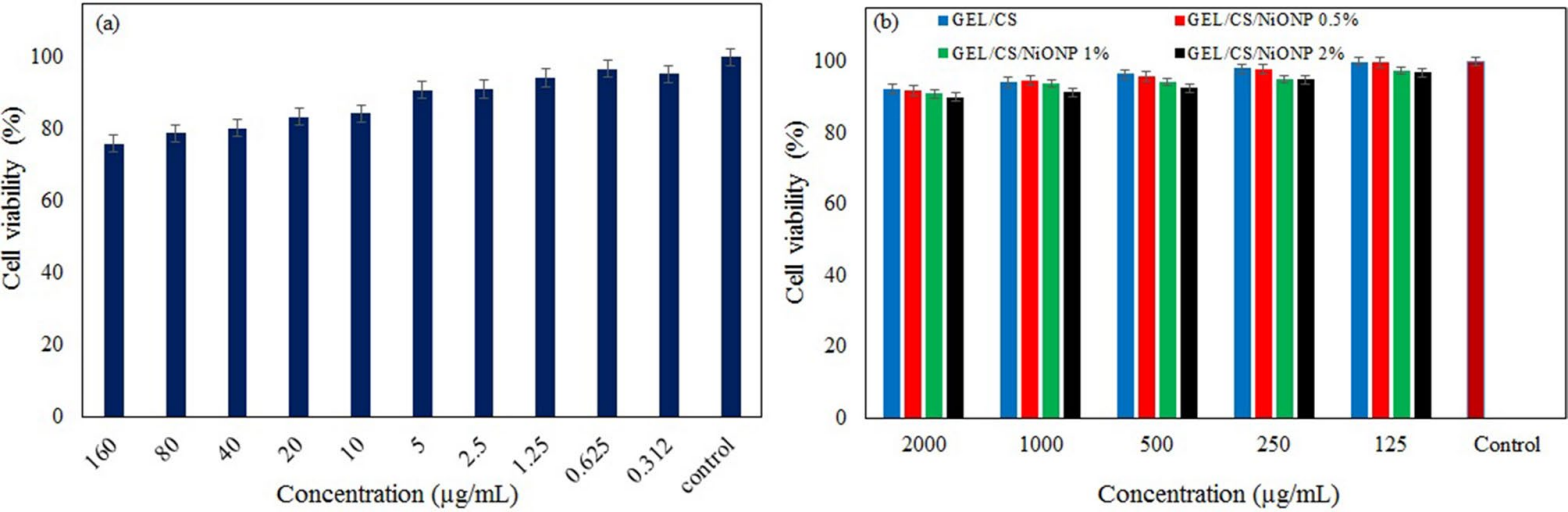
Cell viability of NIH3T3 cells in contact with NPs (**a**) and bio-nanocomposite with various percentages of NiONPs (**b**).

Human skin is the first place that comes into contact with food packaging materials. For this reason, the toxicity of chemicals in packaging materials must be determined. Therefore, skin fibroblast cells are used to evaluate the toxicity of these substances. The toxicity of GEL/CS nanocomposite films and NiONPs was measured on NIH3T3 fibroblast cells, and cell viability was considered as a measurement parameter. The viability of NIH3T3 fibroblast cells in contact with NPs at different concentrations shows (Fig. 3a) that the averaged cell viability at concentrations of 160 µg/mL and 0.312 µg/mL is 78.5% and 95.4%, respectively, and thus the NPs have no toxicity at low concentrations. Figure 3b shows the viability of NIH3T3 cells in contact with GEL/CS films containing different percentages of NPs in the concentration range of 2000-125 µg/mL. From the comparison of Fig. 3(a and b), it can be concluded that the toxicity of the NPs is reduced by addition to the polymer films because the polymer chains prevent direct interaction between the NPs and the cells. These observations are consistent with those reported in the literature^44^. There is a kind of synergistic effect between the NPs and the polymer chains, which reduces the toxicity of the NPs. Considering that the viability of NIH3T3 cells is above 88% in all cases, bio-nanocomposite films of GEL/CS/NiONPs are not toxic to NIH3T3 cells and are recommended for food packaging.

## Conclusions

Biodegradable packaging with antibacterial properties is nowadays highly appreciated to extend the shelf life of food products and reduce their spoilage. In this study, a biocompatible nanocomposite film (GEL/CS/NiONPs) with different percentages of NPs (0.5%, 1% and 2%) was prepared by casting. The analysis results of FTIR, TGA and XRD confirmed the formation of hydrogen bonds between the NPs and the polymer chains, good thermal stability and crystalline structure, respectively. In the FESEM results, the NPs were found to be monodispersed up to 1% throughout the nanocomposite. As a result, the mechanical strength and water resistance of these bio-nanocomposite films were improved. In addition, they exhibited good antimicrobial properties, e.g., they were more active against Gram-positive bacteria than Gram-negative bacteria, and they were not toxic to fibroblast cells. In conclusion, GEL/CS/NiO 1% film was selected as the optimal film due to its excellent thermal, mechanical, barrier and antibacterial properties, and is therefore recommended for food packaging.

## Methodology

### Materials

Low molecular weight chitosan [50000–190000 Da, degree of deacetylation (75–85%)], gelatin (type B, from bovine skin), nickel (II) nitrate hexahydrate (Ni(NO_3_)_2_.6H_2_O) were purchased from Sigma-Aldrich(America). Sodium hydroxide (NaOH), acetic acid glacial (99.9%), calcium sulfate (CaSO_4_), calcium nitrate (Ca(NO_3_)_2_), potassium sulfate (K_2_SO_4_), ethanol (96%), glycerol, dimethyl sulfoxide (DMSO) were purchased from Merck (Germany). Roswell Park Memorial Institute (RPMI 1640), Fetal bovine serum (FBS), Penicillin–Streptomycin (100x), Trypsin- EDTA(1x), 3-(4,5-dimethylthiazol-2-yl)-2,5-diphenyltetrazolium bromide (MTT) were purchased from BioIdiea Co.(Iran). The Standard strains of *E. coli* (ATTC 25922), *S. aureus* (ATTC 25923), and mouse NIH/Swiss embryo (NIH3T3) were purchased from Institute Pasteur Iran.

### Nanoparticles synthesis

The synthesis of NiONPs was carried out by the co-precipitation method in four main steps. First, 20 mL of nickel nitrate solution (0.25 M) was poured into a beaker and 100 mL of 0.5 M sodium hydroxide was added drop by drop to the vessel under vigorous stirring at a temperature of 60°C. The solution was stirred under these conditions for 2 h until the color of the solution changed from pale green to emerald green and settled in the vessel. Second, the sediment was washed four times with pure deionized water and ethanol, each turn on filter paper to wash away unreacted precursors. Third, the sediment was placed in an oven at 80 ˚C for 3 h to dry. After 3 h, the dried sediment should be pounded in a mortar. Fourth, the obtained powder was poured into a porcelain crucible and calcined at a temperature of 400 ˚C for 4 h. The calcined powder is black^20^.

### Films preparation

Bio-nanocomposite films were prepared in three steps: Preparation of chitosan and gelatin solutions, mixing of the film components, and drying of the films. Thus, first, 1 g of chitosan was stirred in distilled water containing 1% v/v acetic acid on a stirrer for 1 h at room temperature (e.g., 25 ˚C), consequently, 1% and 2% w/v chitosan solutions were prepared. On the other hand, a 1% w/v gelatin solution was prepared by stirring 0.35 g gelatin in distilled water at 37 °C for 4 h. Second, a 1:1 ratio of GEL/CS 2% polymer solution was obtained by mixing the respective solutions, and then different percentages of NiONPs (e.g., 0.5%, 1% and 2%) were added separately to the mixtures and stirred for 1 h, after which 30% w/w glycerol based on the original dry polymers (e.g., GEL and CS) was added to each mixture and then stirred for 30 min. Third, 15 mL of the mixture was poured into an 8 cm diameter polystyrene plate and placed on a flat surface to dry at laboratory temperature. After drying, the films were separated from the plate and stored a zippered plastic bag^33^.

### Characterization techniques

The size of NPs was measured with a scanning electron microscope (SEM) (model: LEO1430VP; Catlzeiss, MT, Germany). For this purpose, the surface of the NPs must be electrically conductive, so they were coated with a thin layer of gold and then imaged with a voltage of 15 kV. In addition, the morphology of the GEL/CS cross-section was deciphered using the field emission scanning electron microscope (FESEM). The FESEM instrument with the MIRA3|TESCAN|Czech model was used for this analysis. For sample preparation, a piece of the film was immersed in liquid nitrogen and broken manually. Then the films were fixed to a holder with a double-sided carbon adhesive and coated with platinum, making the surface electrically conductive. Finally, electron microscope images were taken by exposing the samples to electron beams accelerated at a differential voltage of 5 kV. To estimate the PDI of nanoparticles and nanocomposite films, DLS method was performed using a DLS/zeta-sizer device (model: SZ-100). One of the best-known techniques for characterizing molecular interactions and chemical functional groups in a sample is Fourier transform infrared spectroscopy (FTIR). Therefore, a series of FTIR samples of the prepared films were prepared and analyzed in the wavenumber range of 400–4000 cm^−1^ using the FTIR spectrometer available in Iran (Perkin Elmer Spectrum 2, USA). X-ray diffraction (XRD) is still the best method for identifying the crystal structure of the films. Therefore, the XRD patterns of the films were recorded using a diffractometer (Rigaku Ultima Iv, Japan) at a voltage of 40 kV copper beam with a nickel filter in the temperature range of 10–80 degrees. Thermogravimetric analysis and Derivative Thermogravimetry (TGA- DTG) was performed to determine the thermal stability of the nanocomposite films. A thermogravimetric analyzer (model: STA6000, Perkin Elmer, Waltham, Massachusetts, USA) was used for this purpose. To perform this analysis, approximately 2.5 mg of the film was placed in the sample chamber of the instrument and the sample was heated at a heating rate of 10 ˚C/min such that the temperature of the sample increased from 30 to 900 ˚C, and simultaneously the weight loss of the samples was measured as a function of temperature.

### Mechanical properties

The prepared bio-nanocomposites with different percentages of chitosan and NPs (e.g., GEL/CS/NiONPs) were placed under tension to test their mechanical properties according to ASTM standards. Accordingly, the mechanical properties such as tensile strength (TS) and elongation at break (EAB) of the above bio-nanocomposites were evaluated using the STM -150 device. The samples were prepared by cutting the films with a size of 10 × 60 mm^2^. Then, these samples were stretched three times per sample at a rate of 1 mm/min using a length gauge of 40 mm^24^.

### Water resistance properties

Film water resistance, film strength to water vapor permeability (WVP), moisture absorption (MA), and water solubility (WS) are considered in this study. All these tests were repeated three times per sample, so that the averaged results can be relied upon to achieve acceptable confidence.

#### Water vapor permeability

The WVP of the films was tested using the ASTM 69-05 standard^19^. According to this standard, special glass vials with an opening diameter of 2 cm and a height of 4.5 cm can be used for WVP determination. First, the films were cut into slices slightly larger than the opening of the vial. The vials were filled with 3 g of anhydrous calcium sulfate at a relative humidity of 0%, then the opening of the glass was sealed with the films. The vials with the entire contents were weighed and placed in a desiccator with a saturated solution of potassium sulfate (relative humidity equal to 97%), which was in an incubator with a temperature of 25 °C. The observation of a small amount of solid potassium sulfate at the bottom of the desiccator is an indication of the formation of a saturated solution. Vials were weighed every 24 h up to 7 days, and weight changes were recorded as a function of time. The slope of the line, weight versus time, was calculated by linear regression. To calculate WVP, the water vapor transfer rate (WVTR) should be determined, which is obtained by dividing the slope of the line (g/day) of each vial by the surface area (m^2^) of the film exposed to water vapor; then WVP (g mm (m^2^ day kPa)^−1^) can be calculated as follows:

1 $${\text{WVP}}\, = \,\frac{WVTR \times X}{{P \left( {R_{1} - R_{2} } \right)}}$$

Where X presents the film thickness (mm), P stands for the saturated water vapor pressure at 25 °C (3.169 kpa) and R_1_ and R_2_ indicate the relative humidity of the desiccator (97%) and vial (0%), respectively.

#### Moisture absorption

The method MA presented by *S. Amjadi*^45^ was used in this study. For this purpose, the films were prepared with the appropriate dimensions (e.g., 2 × 2 cm^2^) and then placed in a desiccator containing calcium sulfate of 0% relative humidity and weighed after 24 h. The films were then placed in a separate desiccator containing a saturated calcium nitrate solution with 55% relative humidity. The desiccator was at a temperature of 25 °C, and the samples were weighed every 24 h for up to 7 days. The calculation of MA was performed using:

2 $${\text{MA }}\left( {\text{\% }} \right){ } = { }\frac{{W_{t} - W_{0} }}{{W_{0} }}\, \times \,100$$

where w_t_ and w_0_ are the final and initial weights, respectively.

#### Water solubility

Film stability in water according to the method of *S. J. Peighambardoust* was carried out for the bio-nanocomposites^46^. For this test, films samples of 2 × 2 cm^2^ were prepared. These samples were placed in an oven at 105 °C for one hour to reach a constant weight (m_0_). Then, the films were deeply immersed in 50 mL of distilled water in a beaker for 24 h. In the next stage, the wet films were dried under the same drying conditions (105 °C) to achieve a constant weight (m_1_). Consequently, the WS (%) was calculated via:

3 $${\text{WS }}\left( {\text{\% }} \right){ } = \frac{{m_{0} - m_{1} }}{{m_{0} }}\, \times \,100$$

m_0_ and m_1_ are the initial and final weights, respectively.

### Antimicrobial test

The disk diffusion method was used to investigate the antibacterial activity of the prepared bio-nanocomposite films against the standard strain of *E. coli* (ATCC25922) and *S. aureus* (ATCC25923). The inoculum of the bacterial strains was prepared from a 24-h culture in tryptic soy broth, and the bacteria were prepared in physiological serum at a concentration of 10^8^ CFU/mL. Subsequently, 100 µl of the suspension was cultured on Mueller–Hinton agar (MHA) medium using a sterile swab. After cultivation, nanocomposite films were prepared in the form of 5 mm diameter disks and exposed to ultraviolet radiation for 15 min for sterilization, and the disks were placed on MHA medium. The plates were incubated at 37 °C for 24 h. After incubation, the area inhibiting bacterial growth around the films was measured^47^.

### Cytotoxicity evaluation

The toxicity of NiONPs, NP-free GEL/CS films, and GEL/CS films with different percentages of NiONPs, including 0.5%, 1%, and 2%, was studied on NIH3T3 mouse fibroblast cells. The viability of the cells was checked by the cytotoxicity method^48^. The toxicity of the films was evaluated in several steps according to the ISO10993-5 standard. First, NIH3T3 cells were cultured in cell flasks in RPMI-1640 medium containing 10% FBS and 1% of the antibiotics penicillin and streptomycins (100X). Then, the flasks were kept in incubators with 95% humidity and 5% CO_2_ at 37 °C for 24 h. Then, about 10^4^ of the cells (e.g., 100 µl) were seeded in RPMI medium into a 96-well plate and incubated for 24 h at 37 °C, 95% humidity and 5% CO_2_. Meanwhile, 4 mg of each film was sterilized under UV irradiation for 30 min and dissolved in DMSO. Then, different dilutions of these stock solutions (e.g., 2000, 1000, 500, and 125 µg/mL) were prepared, and 100 µl of each dilution was added three times to the plates. Note that the control wells in this assay contain cells without treatment. Then, the plates were incubated for 24 h under the above conditions. In addition, nanoparticle solution with a concentration of 1 mg/mL was prepared. Then, different dilutions of it (160 to 0.312 µg/mL) were prepared and 100 µl of each dilution was added to three separate wells. Like the previous samples, these samples were incubated for 24 h under the above conditions. Then, the samples were removed from the wells and 100 μL of MTT was added to each well and incubated for 2 h. Then, the MTT solution was replaced with 100μL DMSO and incubated for 15 min. Subsequently, the OD of the samples was measured using a BIORAD microplate reader (model 680) at a wavelength of 570 nm. Finally, by using:

4 $${\text{Cell viability }}\left( {\text{\% }} \right) = \frac{{A_{Sample} }}{{A_{Control} }}\, \times \,100$$

equation cell viability percentage was calculated.

### Statistical analysis

For statistical analysis of the resulting data, the well-known SPSS software (version 23) was used in this study. Accordingly, Duncan's multiple ranges and ANOVA tests were performed to determine the statistically significant (*P* < 0.05) results and to report the data as mean ± standard deviation (SD).

## Data Availability

The datasets generated during and/or analyzed during the current study are available from the corresponding author (T. Narimani) on reasonable request.
